# Time to treatment for rifampicin-resistant tuberculosis: systematic review and meta-analysis

**DOI:** 10.5588/ijtld.17.0230

**Published:** 2017-11-01

**Authors:** R. Boyd, N. Ford, P. Padgen, H. Cox

**Affiliations:** * Division of Medical Microbiology, University of Cape Town, Cape Town, South Africa; † Division of Tuberculosis Elimination, Centers for Disease Control and Prevention, Atlanta, Georgia, USA; ‡ Centre for Infectious Disease Research, University of Cape Town, Cape Town, South Africa; § College of Global Public Health, New York University, New York, New York, USA; ¶ Institute of Infectious Disease and Molecular Medicine, University of Cape Town, Cape Town, South Africa

**Keywords:** rifampicin-resistant, tuberculosis, time to treatment

## Abstract

**BACKGROUND:**

To reduce transmission and improve patient outcomes, rapid diagnosis and treatment of rifampicin-resistant tuberculosis (RR-TB) is required.

**OBJECTIVE:**

To conduct a systematic review and meta-analysis assessing time to treatment for RR-TB and variability using diagnostic testing methods and treatment delivery approach.

**DESIGN:**

Studies from 2000 to 2015 reporting time to second-line treatment initiation were selected from PubMed and published conference abstracts.

**RESULTS:**

From 53 studies, 83 cohorts (13 034 patients) were included. Overall weighted mean time to treatment from specimen collection was 81 days (95%CI 70–91), and was shorter with ambulatory (57 days, 95%CI 40–74) than hospital-based treatment (86 days, 95%CI 71–102). Time to treatment was shorter with genotypic susceptibility testing (38 days, 95%CI 27–49) than phenotypic testing (108 days, 95%CI 98–117). The mean percentage of diagnosed patients initiating treatment was 76% (95%CI 70–83, range 25–100).

**CONCLUSION:**

Time to second-line anti-tuberculosis treatment initiation is extremely variable across studies, and often unnecessarily long. Reduced delays are associated with genotypic testing and ambulatory treatment settings. Routine monitoring of the proportion of diagnosed patients initiating treatment and time to treatment are necessary to identify areas for intervention.

## Abstract (FR)

**CONTEXTE:**

Pour réduire la transmission et améliorer le devenir des patients, un diagnostic et un traitement rapides de la tuberculose résistante à la rifampicine (TB-RR) sont requis.

**OBJECTIF:**

Réaliser une revue systématique et une méta-analyse évaluant le délai de traitement de la TB-RR et la variabilité en fonction de la méthode de test de diagnostic et du mode de prestation du traitement.

**SCHEMA:**

Les études (2000–2015) rapportant des délais de mise en route du traitement de deuxième ligne ont été sélectionnées sur PubMed et dans des résumés de conférence publiés.

**RESULTATS:**

A partir de 53 études, 83 cohortes (13 034 patients) ontété incluses. Dans l'ensemble, le délai moyen pondéré de traitement depuis le recueil d'échantillons a été de 81 jours (IC95% 70–91), plus court en traitement ambulatoire (57 jours, IC95% 40–74) qu'hospitalier (86 jours, IC95% 71–102). Le délai de traitement a été plus court avec le test de sensibilité génotypique (38 jours, IC95% 27–49) plutôt que phénotypique (108 jours, IC95% 98–117). Le pourcentage moyen de patients diagnostiqués mis sous traitement a été de 76% (IC95% 70–83%, fourchette 25–100%).

**CONCLUSION:**

Le délai de mise en route du traitement de deuxième ligne de TB est extrêmement variable selon les études, et souvent inutilement long. Une réduction des délais est associée à l'utilisation d'un test génotypique et à un traitement ambulatoire. Le suivi de routine de la proportion de patients diagnostiqués mis sous traitement et du délai de traitement est nécessaire pour identifier des domaines d'intervention.

## Abstract (SP)

**MARCO DE REFERENCIA:**

Con el propósito de disminuir la transmisión de la tuberculosis resistente a rifampicina (TB-RR) y mejorar los desenlaces de los pacientes que la padecen, es preciso procurar un diagnóstico temprano y el comienzo rápido del tratamiento.

**OBJETIVO:**

Se llevó a cabo una revisión sistemática con metanálisis de las publicaciones científicas que evaluaban el lapso hasta iniciar el tratamiento de la TB-RR y su variabilidad en función de los métodos diagnósticos y la estrategia de suministro del tratamiento.

**MÉTODO:**

De la base de datos PubMed y los resúmenes de conferencias se escogieron los estudios (publicados del 2000 al 2015) que notificaban el lapso hasta el comienzo del tratamiento antituberculoso de segunda línea.

**RESULTADOS:**

De los 53 estudios examinados, se incluyeron 83 cohortes (13 034 pacientes). La media ponderada global del lapso entre la recogida de la muestra y el comienzo del tratamiento fue 81 días (IC95% de 70 a 91) y el intervalo fue más corto con el tratamiento ambulatorio (57 días; IC95% de 40 a 74) que con el tratamiento hospitalario (86 días; IC95% de 71 a 102). El lapso hasta el comienzo del tratamiento fue menor cuando se practicaron pruebas genotípicas de sensibilidad a los medicamentos (38 días; IC95% de 27 a 49) que con las pruebas fenotípicas (108 días; IC95% de 98 a 117). El promedio de los pacientes diagnosticados que iniciaron tratamiento fue 76% (IC95% de 70 a 83; amplitud de 25% a 100%).

**CONCLUSIÓN:**

El lapso hasta el comienzo del tratamiento antituberculoso de segunda línea es extremadamente variable en los diferentes estudios y con frecuencia se prolonga sin necesidad. La disminución del retraso se asoció con los entornos donde se practican las pruebas genotípicas de sensibilidad y el tratamiento ambulatorio. La supervisión sistemática de la proporción de pacientes diagnosticados que comienzan el tratamiento y del lapso hasta su iniciación es primordial con miras a reconocer las actividades que precisan intervención.

MULTIDRUG-RESISTANT TUBERCULOSIS (MDR-TB, defined as TB resistant to both isoniazid and rifampicin [RMP]) is a global health threat.^1^ The World Health Organization (WHO) estimates that 580 000 people developed RMP-resistant TB (RR-TB) globally in 2015, accounting for 250 000 deaths.^2^ RR-TB, including MDR-TB, is more difficult to diagnose and treat than drug-susceptible TB, requiring longer courses of treatment. Globally, less than 30% of estimated RR-TB patients are diagnosed, and fewer are started on appropriate second-line treatment.^3^

For the minority of RR-TB patients who are appropriately diagnosed and receive second-line treatment, delays to treatment initiation are often many months in some settings.^4–9^ Such delays are likely to increase mortality and loss to follow-up while awaiting treatment,^10^,^11^ in addition to potentially poorer treatment outcomes among those who do start treatment.^12^ Long delays to treatment are also likely to contribute substantially to transmission in both community and nosocomial settings.^13–15^ Given that the majority of RR-TB patients in high-burden settings are likely due to direct transmission,^16^ scale-up of diagnosis and rapid initiation of effective treatment are required to improve patient outcomes and reduce ongoing transmission.^17^

A range of health system factors may influence time from first presentation at a health service to treatment initiation, including access to diagnostic services, complicated referral processes and availability of second-line treatment. Before the availability of genotypic drug susceptibility testing (DST), resistance testing relied on culture-based (phenotypic) methods, often taking months to receive results. Increased use of polymerase chain reaction based tests such as line-probe assays (LPAs) and Xpert^®^ MTB/RIF (Cepheid, Sunnyvale, CA, USA) have reduced the laboratory time needed to reach a diagnosis of RR-TB, and therefore should theoretically reduce delays in treatment initiation. Similarly, the provision of community-based treatment, without mandatory admission to hospital, as recommended by the World Health Organization (WHO),^18^ should both increase access to treatment and reduce delays.

We aimed to conduct a systematic review and meta-analysis to assess time to second-line treatment among RR-TB patients and to assess delay in terms of DST methods, access to ambulatory treatment compared to hospital-based treatment, and the proportion of patients who start treatment.

## METHODS

### Search strategy

We followed the Preferred Reporting Items for Systematic Reviews and Meta-Analyses (PRISMA) guidelines.^19^ Using a sensitive search strategy comprised of a combination of MeSH terms and other key terms,^*^ we searched PubMed (including Medline) and Scopus for relevant articles published from 1 January 2000 to 15 July 2015, without language restrictions. We reviewed abstract books from the Union World Conference on Lung Health from 2010 to 2014 for studies that may have been completed but not yet published. Additional articles were identified from bibliographies of articles that underwent full-text review.

### Study selection

We included studies reporting time to second-line treatment initiation in RR-TB patients, including MDR-TB and extensively drug-resistant TB (XDR-TB, defined as MDR-TB plus resistance to any fluoroquinolone and at least one of the three second-line anti-tuberculosis injectable drugs, capreomycin, kanamycin, or amikacin). Only studies reporting mean or median times to treatment and standard deviations (SDs) (or with available data allowing calculation of these figures) were eligible to be included in the meta-analysis. Case reports and studies with small sample size (<10 persons) were excluded. Our intention was not to perform a traditional quality assessment, but to set inclusion and exclusion criteria to identify as many comparable studies as possible while also avoiding low-quality studies. Two authors (RB, HC) independently reviewed titles and abstracts to identify potentially eligible articles, which then underwent full review to determine final eligibility status, with the same two authors dividing this effort with overlap. Any discrepancy or uncertainty was resolved by consensus. Abstracts and/or articles in languages other than English were translated. Additional articles published after the defined dates were included only if identified through abstracts published during the initial defined time period.

### Data extraction

Two authors (RB, HC) extracted data for each cohort described in the included articles. The following information was sought: study year(s), country, sample size, study design, time to treatment definition, mean and median time to treatment, DST method, model of treatment provision and proportion of patients starting treatment. Attempts were made to contact authors of eligible or potentially eligible studies to provide missing data or clarifications. Study quality and potential bias were assessed by reviewing study design, primary outcomes and availability of adequate time to treatment data.

### Definitions

Studies were grouped according to definition of time to treatment. The main categories were defined as either time from date of specimen collection or date of diagnosis. Date of diagnosis included a range of definitions given, including date of result available or received by clinician, or defined simply as date of diagnosis (unclear definition). Studies that used other definitions of time to treatment are listed in the Appendix Table^4–6^,^8^,^11^,^20–65^^†^, but were not included in grouped analyses. Diagnostic methods were defined as phenotypic if DST methods included liquid or solid culture methods, and genotypic if based on any genotypic method, such as LPA or Xpert, even if conducted after a positive culture. The model of treatment provision was defined as hospital-based if patients were hospitalized or relocated close to a hospital to initiate treatment, and was defined as ambulatory if patients were able to receive treatment on an ambulatory basis during the full course of treatment.

### Data analysis

The primary outcome was mean time to treatment. Where this was not reported, means and SDs were estimated based on methods described in Wan et al.^66^ We performed both within-study comparative meta-analysis as well as analyses across studies to describe the impact of varying DST methods and models of treatment provision. For within-study analysis, any study was eligible to be included, irrespective of definition of time to treatment used, provided they included two cohorts comparing at least one variable of interest. Weighted mean differences (WMDs) and corresponding 95% confidence intervals (95%CIs) were calculated to standardize the results of the studies to a uniform scale and to indicate the size of the intervention effect in each study relative to the variability observed in that study. For the across-study analyses, pooled data were stratified by time from specimen collection or from diagnosis; weighted means and corresponding 95%CIs were calculated. Because statistical tests for heterogeneity are not reliable for pooled proportions,^67^ heterogeneity was assessed by visual inspection of forest plots, and changes in mean time to treatment over time assessed through meta-regression. All analyses were conducted using STATA version 13.0 (StataCorp, College Station, TX, USA).

## RESULTS

**Figure 1:**
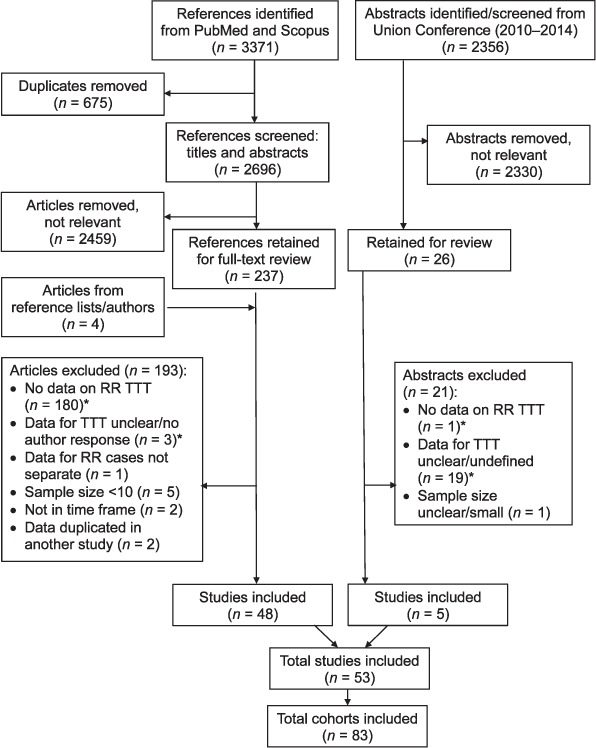
Study selection process flowchart. RR =rifampicin-resistant; TTT = time to treatment.

From a screen of 1768 articles and 2356 conference abstracts, a total of 48 published studies and 5 abstracts were included in the systematic review (Figure 1). Many studies included more than one patient cohort; these are reported separately. The Appendix Table describes study characteristics, time to treatment definitions, mean and median time to treatment and the proportion of diagnosed patients who were treated.

Studies were from 21 countries, and included 83 cohorts, ranging in sample size from 10 to 1063, with a total sample size of 13 034. Twenty-three cohorts were classified as ambulatory, and 58 were hospital-based (2 indeterminable). Phenotypic DST was used for 53 cohorts; 29 used genotypic DST, 12 of which incorporated Xpert (partially or fully) (1 indeterminable). The proportion of diagnosed patients who initiated treatment was reported for 31 cohorts. Study design was prospective for 19 (23%) cohorts and retrospective for 64 (77%) cohorts. Time to treatment was a primary outcome for 26/53 (49%) studies, representing 47/83 (57%) cohorts.

### Time to treatment

Mean time to treatment was reported for 30 cohorts and calculated for the remaining 53 cohorts. There were insufficient data available to calculate SDs for seven cohorts; these are listed in the Table but not included in the analyses. Time to treatment was most commonly reported as time from specimen collection (38 cohorts), followed by time from diagnosis (28 cohorts; Appendix Table).

Mean and median times to treatment from specimen collection ranged from respectively 9 days to 10 months and 8 days to 9 months. Among the 38 cohorts with time to treatment measured from specimen collection, the weighted mean time to treatment was 81 days (95%CI 70–91, range 9–301). Among the 24 cohorts with time to treatment measured from diagnosis, the weighted mean time to treatment was 59 days (95%CI 50–68, range 2–909).

### Model of treatment provision

**Figure 2:**
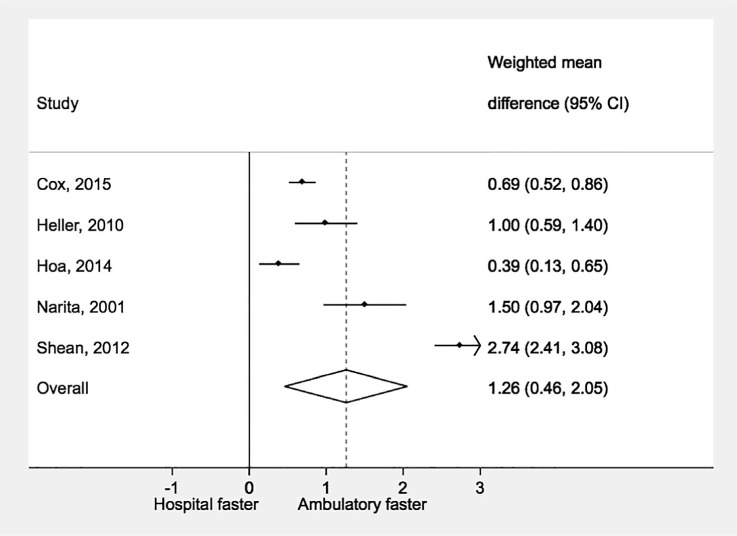
Time to treatment initiation by model of treatment provision. WMD =weighted mean difference; CI = confidence interval.

Five studies were included in the within-study comparison of ambulatory vs. hospital-based treatment provision (Figure 2). All five studies reported faster time to treatment for patients under ambulatory treatment compared to hospital-based treatment; the pooled difference across all studies was significantly in favor of ambulatory treatment (WMD 1.26, 95%CI 0.46–2.05).

There were seven (1763 patients) cohorts treated under ambulatory-based models of care and 29 (4250 patients) under hospital-based treatment with time to treatment from specimen collection. Mean time to treatment with ambulatory treatment was 57 days (95%CI 40–74, range 17–122) compared to 86 days (95%CI 71–102, range 9–301).

### Drug susceptibility testing methods

**Figure 3:**
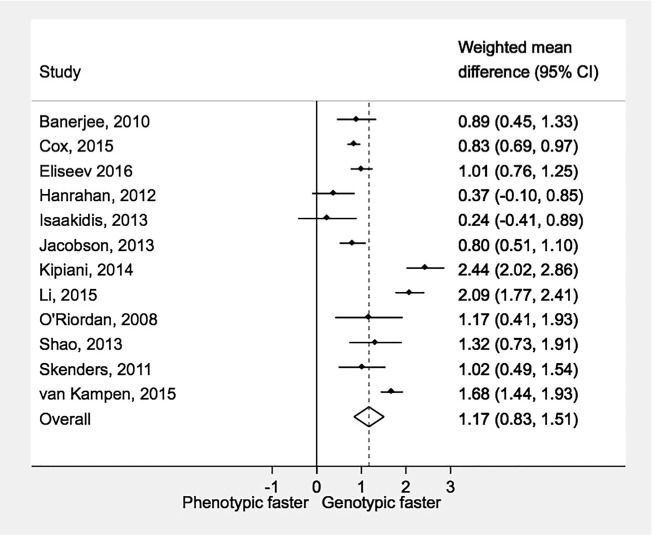
Time to treatment initiation by laboratory drug susceptibility testing methods. WMD = weighted mean difference; CI = confidence interval.

Twelve studies were included in the within-study comparison of DST methods (Figure 3). All studies consistently reported a shorter time to treatment with genotypic vs. phenotypic DST; the pooled difference across all studies was significantly in favor of genotypic DST (WMD 1.17, 95%CI 0.83–1.51). There were 14 (3842 patients) cohorts using genotypic DST and 23 (2460 patients) cohorts with phenotypic DST reporting time to treatment from specimen collection. Mean time to treatment was significantly lower with genotypic DST: 38 days (95%CI 26–49, range 9–94) vs.108 days (95%CI 98–117, range 52–301) for phenotypic DST.

### Time to treatment by year of cohort

**Figure A.1:**
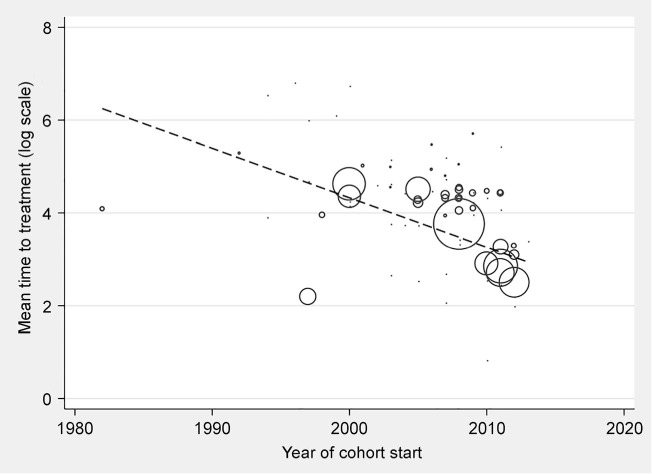
Trends in mean time to treatment initiation over time.

Among cohorts with time to treatment measured from specimen collection, the mean time to treatment decreased over time (β-coefficient −3.13, 95%CI −5.09 to −1.18, *P* =0.002; Appendix Figure A.1). The weighted mean time to treatment from specimen collection before 2010 was 98 days (95%CI 85–111, range 9–301) compared to 39 days (95%CI 28–50, range 12–87) for 2010 or later.

### Time to treatment by proportion initiating treatment

**Figure A.2:**
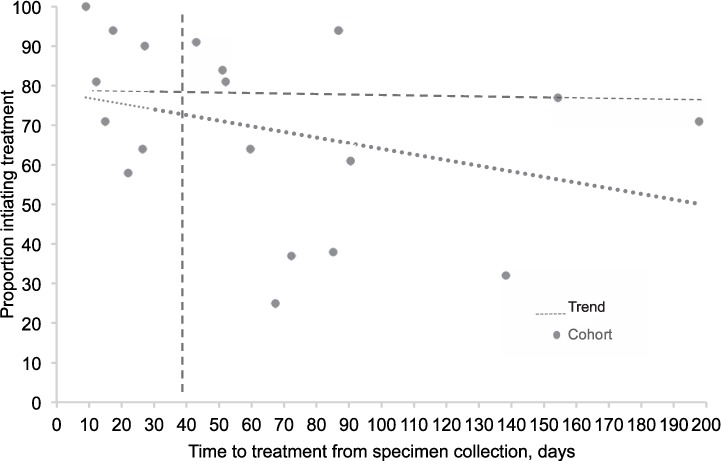
Time to treatment initiation by proportion initiating treatment. Dotted lines at 80% and 30 days highlight four cohorts with both high proportion initiating treatment and short time to treatment, to represent best practice.^4^,^31–33^

The mean percentage of diagnosed patients initiating treatment (reported for 31/83 cohorts) was 76% (95%CI 70–83, range 25–100; Appendix Table). Appendix Figure A.2 compares mean time to treatment to the proportion initiating treatment for the 19 cohorts reporting time to treatment from specimen collection. The upper-left shaded portion represents cohorts with a mean time to treatment of ⩽30 days and at least 80% of diagnosed patients initiating treatment to represent best practice; only four cohorts,^4^,^30^,^32^,^33^ representing 458/3286 (14%) patients included in the analysis, fell into this category. All four cohorts used genotypic DST; model of treatment provision was ambulatory for two cohorts, hospital-based for one and not reported for one.

## DISCUSSION

Delays in initiation of second-line treatment can negatively impact clinical and public health outcomes. Even reductions of several weeks or months are likely to significantly impact community transmission^16^ and are likely to improve patient outcomes.^12^,^39^ This systematic review and meta-analysis has shown that time to treatment is extremely variable and often lengthy. Overall, the average time to treatment from specimen collection was 2.5 months, with a trend towards reduction in delay in more recent years. This is consistent with advances in RR-TB diagnosis and treatment, and potentially reflects greater recognition of the need to initiate treatment sooner to improve patient outcomes and reduce ongoing risk of transmission. Genotypic DST methods and ambulatory-based models of care both contributed to shorter times to treatment.

Molecular testing methods result in more rapid laboratory turnaround times,^12^,^68–70^ and are therefore likely to reduce time to treatment. This was confirmed in our analysis, with genotypic testing resulting in significantly shorter time to treatment than phenotypic methods; our findings are consistent with the results of a randomized trial^71^ and a retrospective cohort study published after our search was concluded.^72^ Xpert is of particular interest due to the feasibility of testing in peripheral laboratories,^73^,^74^ potentially reducing reliance on transport and resulting in more rapid communication of results. Studies that have implemented faster molecular DST show lower mortality and loss to follow-up, and therefore a higher proportion of patients starting treatment.^37^ Rapid DST has also been shown to reduce treatment failure^57^ and result in higher treatment success.^39^ However, currently available genotypic methods are restricted by the number of drugs than can be tested, often resulting in continued reliance on phenotypic DST for second-line drugs.

Ambulatory second-line treatment can result in treatment outcomes similar to those of hospital-based treatment,^75^ and can lead to higher proportions of patients initiating treatment.^4^,^30^,^43^ Our review complements these positive findings, providing evidence that ambulatory treatment results in shorter time to treatment than hospital-based treatment. Patients receiving treatment in hospital-based settings may experience further delays due to the preparation needed to be admitted to the hospital; these may include referral processes, informing family and work, making arrangement for the care of children and other home responsibilities, and actually traveling to the hospital.

We identified a wide range in delay across studies, particularly among cohorts with hospital-based models of care as well as cohorts with phenotypic DST. The authors of the main studies with lengthy times to treatment refer to prolonged referral processes^7^ and the use of phenotypic DST methods.^32^ Although reduced delays are seen with both genotypic DST and ambulatory treatment provision, several more recent studies show times to treatment of >1.5 months.^4^,^6^,^25^ Studies report delays in reporting results to clinics and in contacting patients as potential contributing factors.^26^,^30^ Programmatic factors such as sample transport and results communication could be improved.^76^

Time to initiation of second-line treatment needs to be considered in terms of the proportion of diagnosed patients who actually start treatment. Several studies reported relatively rapid times to treatment (<30 days), but with <70% of diagnosed patients starting on treatment.^21^,^37^,^51^ These studies highlight the need to assess areas of improvement along the entire diagnosis and treatment cascade for RR-TB, from diagnosis of TB, to identification of drug resistance, to treatment initiation and finally, to treatment success.

Our systematic review has identified several limitations in the current evidence base. First, the definitions of time to treatment were not reported clearly or consistently across several studies, and were grouped into categories described in the Table for ease of analysis. Studies reporting time to treatment from specimen collection can provide a clearer picture of delays caused by various elements in health care systems, including specimen transport, diagnostic delays, reporting of results, patient notification and referral. However, several delays could have occurred before sending a specimen for DST, including patient-level delays in seeking treatment and restricted access to DST. Without universal access to DST, patients may be treated first for drug-susceptible TB and only be offered DST upon failure of treatment. Second, neither time to treatment nor the proportion of diagnosed patients initiating treatment were primary outcomes for many of the studies in this analysis. This contributes to unclear definitions and also uncertainty introduced through calculation of means and SDs. The inconsistency in reporting the proportion of patients initiating treatment (only reported for <40% of cohorts) may also skew the time to treatment data. Third, there may be other factors influencing time to treatment that were not reported by the studies and could not be assessed in our analyses, including decentralized laboratory services, availability and accessibility of treatment services, and inclusion of migratory populations. Fourth, due to lack of data, authors were not able to stratify analysis by Xpert and LPA. Another important limitation to the conduct of this review is the limited number of databases that were searched. One study in this analysis is a randomized control trial, and we acknowledge that this may introduce bias, as additional delays may be caused by the randomization process; it is important to note that this study is not included in the majority of analyses for this review, i.e., those that measure time to treatment from sputum collection, and it therefore has little impact on the primary findings. Furthermore, 77% of the cohorts in this review are from retrospective studies, and we acknowledge risk of bias with retrospective study design. Finally, as with any systematic review, there may be publication bias.

The proportion of diagnosed RR-TB patients who initiate treatment and the time to second-line treatment are important indicators of programmatic performance. While the proportion of the estimated global burden of RR-TB that receives treatment is gradually increasing, there is still much room for improvement.^2^ The WHO End TB Strategy calls for integrated patient-centered care and prevention, including universal DST and treatment of all people with RR-TB; bold policies and supportive systems, including political commitment and engagement of communities; and intensified research and innovation.^77^ Such interventions and commitment should contribute to reducing the diagnostic and treatment gaps, and treatment delays. Routine monitoring and reporting of the proportion of patients initiating treatment and time to treatment, ideally measured from specimen collection to highlight most delays, are needed to identify gaps and areas for intervention.

## Figures and Tables

**Table:**
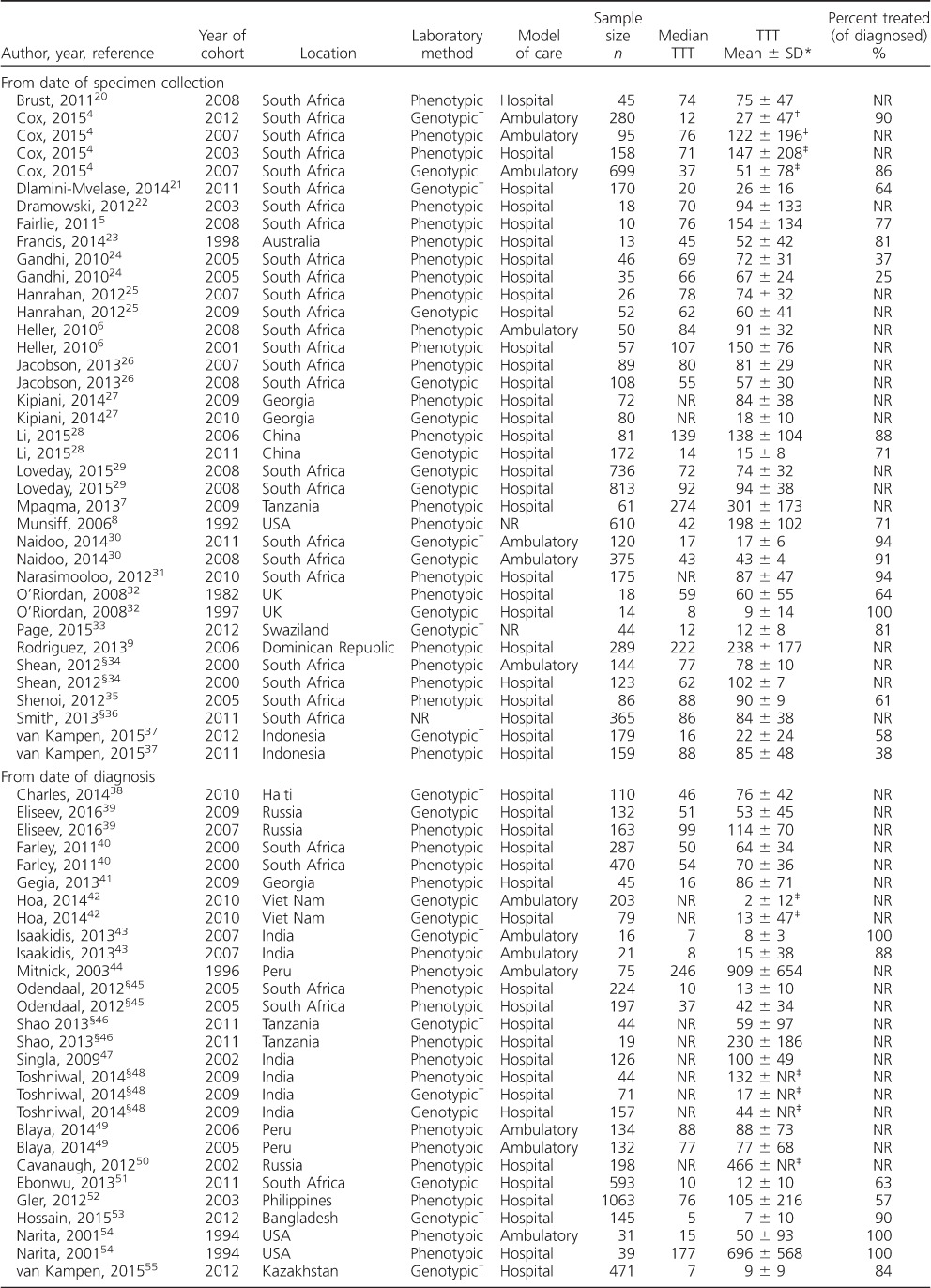

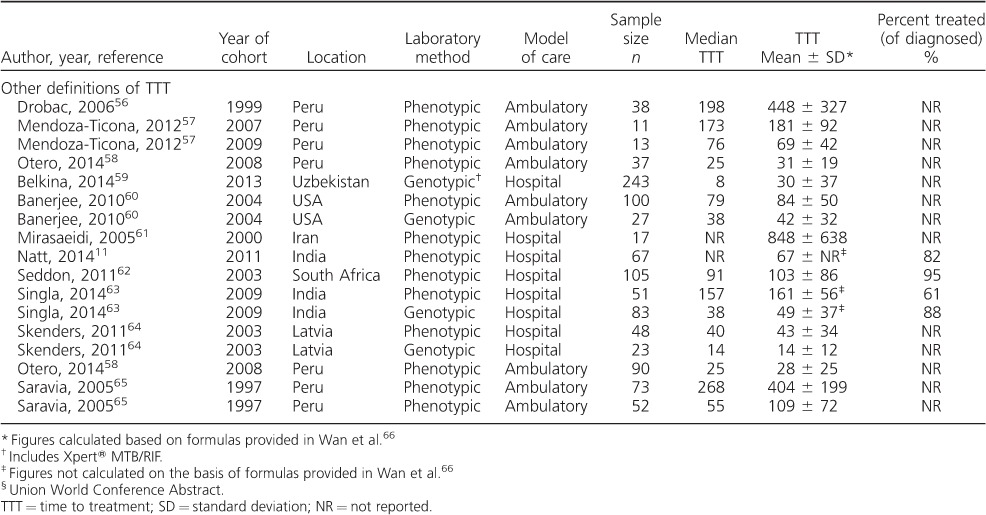
Study characteristics and results

## Footnotes

^*^ The study protocol is available on request from the corresponding author.

^†^ The appendix is available in the online version of this article, at http://www.ingentaconnect.com/content/iuatld/ijtld/2017/00000021/00000011/art00014
